# Mānuka Oil vs. Rosemary Oil: Antimicrobial Efficacies in Wagyu and Commercial Beef against Selected Pathogenic Microbes

**DOI:** 10.3390/foods12061333

**Published:** 2023-03-21

**Authors:** Ramandeep Kaur, Lovedeep Kaur, Tanushree B. Gupta, John Bronlund

**Affiliations:** 1 School of Food and Advanced Technology, Massey University, Palmerston North 4442, New Zealand; 2 Riddet Institute, Massey University, Palmerston North 4442, New Zealand; 3 Food System Integrity Team, Hopkirk Research Institute, AgResearch Ltd., Palmerston North 4472, New Zealand

**Keywords:** antimicrobial agents, beef, food safety, kānuka oil, mānuka oil, meat products, natural preservatives, rosemary oil, wagyu

## Abstract

Essential oils possessing antimicrobial characteristics have acquired considerable interest as an alternative to chemical preservatives in food products. This research hypothesizes that mānuka (MO) and kānuka (KO) oils may possess antimicrobial characteristics and have the potential to be used as natural preservatives for food applications. Initial experimentation was conducted to characterize MOs (with 5, 25, and 40% triketone contents), rosemary oil (RO) along with kanuka oil (KO) for their antibacterial efficacy against selected Gram-negative (*Salmonella* spp. and *Escherichia coli*), and Gram-positive (*Listeria monocytogenes* and *Staphylococcus aureus*) bacteria through disc diffusion and broth dilution assays. All MOs showed a higher antimicrobial effect against *L. monocytogenes* and *S. aureus* with a minimum inhibitory concentration below 0.04%, compared with KO (0.63%) and RO (2.5%). In chemical composition, α-pinene in KO, 1, 8 cineole in RO, calamenene, and leptospermone in MO were the major compounds, confirmed through Gas-chromatography-mass spectrometry analysis. Further, the antimicrobial effect of MO and RO in vacuum-packed beef pastes prepared from New Zealand commercial breed (3% fat) and wagyu (12% fat) beef tenderloins during 16 days of refrigerated storage was compared with sodium nitrate (SN) and control (without added oil). In both meat types, compared with the SN-treated and control samples, lower growth of *L. monocytogenes* and *S. aureus* in MO- and RO- treated samples was observed. However, for *Salmonella* and *E. coli*, RO treatment inhibited microbial growth most effectively. The results suggest the potential use of MO as a partial replacement for synthetic preservatives like sodium nitrate in meats, especially against *L. monocytogenes* and *S. aureus*.

## 1. Introduction

Beef paste is an important material used in the preparation of meat products such as sausages, hamburgers, meat pies, meatballs, and dried meat slices, owing to its high nutritional value and convenient use. It is more susceptible to microbial growth and oxidative reactions, as it promotes the distribution of microbes and oxygen by disrupting muscle membrane integrity and increasing surface area [1]. It has been reported that Salmonella Typhimurium (DT104) incidences have been associated with meat paste, chicken, pork sausages, and several other food products like unpasteurized milk products and fresh apple cider. These products represented around 38% of human salmonellosis in 2000 in Canada [2,3]. Pathogenic microbes such as *Listeria monocytogenes*, *Escherichia coli*, and *Staphylococcus aureus* may be found in different meat products, including beef, pork, poultry, lamb, and mutton [4]. The predominant meat spoilage microbes are Actinobacteria, Firmicutes, and Proteobacteria, in which Carnobacterium spp., Brochothrix thermosphacta, and lactic acid bacteria are the most ubiquitous on raw and packaged meat products and form a large part of the core meat community on meats [4,5,6,7]. These products require the addition of antimicrobials or preservatives to prevent the growth of food pathogenic and spoilage bacteria. Chemical preservatives like sodium/potassium nitrate and nitrites have long been used in raw and processed meats to alleviate lipid oxidation and pathogenic and spoilage microbes’ growth [8,9,10,11]. These are added to processed meats such as ham, bacon, raw meats, and sausages (uncooked raw, semi-smoked, frankfurter, boiled-type, liver pâtě, and mortadella) to prevent the growth of *Clostridium botulinum* and other pathogenic microbes [10]. In addition, nitrates/nitrites retard the spoilage oxidative reactions and ameliorate the colour and flavour of meat products. However, besides the desirable effects, the consumption of nitrates/nitrites can form endogenous N-nitrosamine, a few of which have been reported as carcinogenic [8,12,13,14,15]. Hence, driving the need to use preservatives of natural or plant-based origin in meat products. In this context, plant essential oils, which have been reported to possess antimicrobial activities against microorganisms associated with food spoilage, could be a promising alternative to chemical preservatives [16,17]. Various plants’ essential oils, such as thyme, clove, oregano, ginger, rosemary, and basil, have been reported for their use in fresh, processed, minced, and cooked meat products [18,19,20,21,22,23]. However, no research report is available on the usage of mānuka oil (MO) as a preservative in meat and meat products, even though some studies on this oil’s in vitro antimicrobial potential have been reported [24,25,26,27]. 

*Leptospermum scoparium* (mānuka) and *Kunzea ericoides* (kānuka) are New Zealand’s indigenous plants belonging to the *Myrtaceae* family. Like the Australian tea tree oil, essential oils obtained from these plants (MO and kānuka oils (KO)) are known as New Zealand tea tree oils. The antimicrobial potential of MO against a wide range of microbes has been reported in the literature [25,28]. The existing research documented that the powerful antimicrobial characteristics of the mānuka oil are due to the presence of triketones [28]. As per the available literature, the antimicrobial effect of KO could be attributed to the monoterpenes, especially alpha-pinene [24].

It has been reported that antimicrobial efficacies of essential oils are lower in food matrices than in vitro systems (in broth media), possibly due to the interactions of the oil with food constituents [29]. A probable reason could be the lower water content of foods than the laboratory media, which may hamper the progress of antimicrobial agents like essential oils toward the target site in the microbial cell. Additionally, levels of fat present in food may dissolve the essential oils; thereby, they may be relatively less available to make contact with microbes existing in the aqueous phase of food. The solubilizing effect of lipids in foods may influence the bioactive and antimicrobial activity of essential oils [30]. For instance, the study of Singh, et al. [31] reported that the incorporation of thyme oil could not show an appropriate effect in full-fat hotdogs, whereas, in low and zero-fat hotdogs, it reduced the *Listeria monocytogenes* numbers. Similarly, Wang, et al. [32] reported a decreased antimicrobial activity of carvacrol in regular beef (12% fat) than in lean beef (5% fat) due to the absorption of carvacrol in the fat of regular ground beef. 

Black Japanese cattle, also known as wagyu, is an extensively marbled (intramuscular fat) beef product renowned for its unique flavour and tenderness [33]. The higher intramuscular fat content and unsaturated fatty acid composition of wagyu beef than Angus and other beef breeds have been reported in various studies [33,34,35]. 

Due to increased consumer consciousness, there is a growing demand for chemical additive-free meat products. Various in vitro studies have shown that MO and KO possess antimicrobial characteristics. In light of this, this research hypothesizes that MO and KO may be used as natural alternatives to synthetic antimicrobial agents. Preliminary experimentation involving the characterization and comparison of MO (with 5, 25, and 40% triketone content), KO with a commonly used natural preservative, and rosemary oil (RO) [36], for their antibacterial efficacies against selected pathogenic Gram-positive, (*Listeria monocytogenes* (*L. monocytogenes*) and *Staphylococcus aureus* (*S. aureus*)), and Gram-negative, (*Salmonella* spp. (*Salmonella*) and *Escherichia coli* (*E. coli*)), microbes, was performed. Further, this study examined the antimicrobial potential of MO and RO as an alternative to chemical preservatives like sodium nitrate (SN) in low (3%)- and high (12%)-fat meat products. KO was not selected to test in meat pastes due to its lower antimicrobial activity than MO and RO. 

## 2. Materials and Methods

### 2.1. Materials

The mānuka oils with different triketone contents, i.e., 5, 25, and 40%, and kanuka oil, were kindly provided by Tairawhiti Pharmaceuticals Ltd. (Te Araroa, New Zealand), while the rosemary oil was bought from “Now Foods” (Auckland, New Zealand). The producer provided the blends of 5, 25, and 40% manuka oil triketones (a mixture of three compounds, i.e., leptospermone, isoleptospermone, and flavesone) combined with other compounds, including sesquiterpenes. All oil samples were stored at 4 °C in amber-coloured glass bottles. The bottles were packed in black plastic bags to prevent the effect of temperature and light on the volatiles of the oils. Each oil sample was dissolved in dimethyl sulfoxide (DMSO) to test in vitro antimicrobial effect of oils against microbial growth. 

The vacuum-packed meat samples of grass-fed wagyu and commercial breed beef tenderloins were purchased from Gourmet Butchery, Napier (New Zealand). The meat samples were stored at −20 °C in a freezer and thawed overnight before the analysis. In this paper, mānuka oil with 5%, 25%, and 40% triketone contents have been referred to as mānuka oil 1 (MO 1), mānuka oil 2 (MO 2), and mānuka oil 3 (MO 3), respectively.

### 2.2. Chemical Composition (Using Gas Chromatography-Mass Spectrometry) Analysis of Essential Oils

The gas chromatography-mass spectrometry (GC-MS) analysis of all oil samples was performed using the method of Van Vuuren, et al. [37]. The TG-5MS (Thermo Fisher, Waltham, MA, USA) GC column (30 m × 0.25 mm × 0.25 µm) was used in this analysis. The essential oil samples were injected using a split ratio of 100:1 and an oven temperature of 220 °C. The initial temperature used was 60 °C (for 10 min), then rising to 220 °C (at a rate of 4 °C/min), held for 10 min, and again increasing to 240 °C (at a rate of 1 °C/min). The detector conditions, such as temperature and ionization mode, were set at 250 °C and electron impact, respectively. The chemical components were identified by comparing the obtained peaks with the mass spectra library (NIST 05). 

### 2.3. Thermogravimetric Analysis (TGA) Analysis of Essential Oils

Thermogravimetric analysis (TGA) was conducted with a thermal analyzer (TGA, model STA 449 F5 Jupiter) to check the thermal stability of mānuka, kanuka, and rosemary oils. Each oil sample of 10 mg was heated from 30 °C to 300 °C with a heating rate of 10 °C/min. The heating curve data were analyzed using the NETZSCH ASC software (NETZSCH, Selb, Germany). 

### 2.4. Determination of Antimicrobial Potential of Oils

#### 2.4.1. Bacterial Strains and Media

*E. coli* (ATCC 25922, NZRM 916) and *Salmonella* (NZRM 4030) were grown and maintained in nutrient broth, and *S. aureus* (ATCC 25923, NZRM 917) and *L. monocytogenes* (NZRM 4230) were grown in tryptic soy broth (TSB) for 24 h at 37 °C. The selective agar plates used were brilliant green modified agar for Salmonella, eosin methylene blue (EMB) for *E. coli*, oxford’s agar for *L. monocytogenes*, and Baird Parker’s agar plates for *S*. *aureus*.

#### 2.4.2. Disc Diffusion Assay

The disc diffusion assay of essential oils diluted in 0.01% dimethyl sulfoxide (DMSO) (essential oil at 5% concentration) was performed according to the method described by Jeong, et al. [38], with some modifications. We checked that DMSO at this concentration had no antibacterial effect against all tested microbes (data not shown). Its effect against *L. monocytogenes* is presented in Supplementary Figure S2, and against other microbes, the data for this is not shown. Firstly, the overnight-grown bacterial cultures were adjusted to 0.5 McFarland turbidity standard (around 10^8^ cfu/mL), and the bacterial suspension was uniformly swabbed on Mueller Hinton agar plates using sterile cotton swabs. The sterilized paper discs (about 6 mm diameter) were placed in the centre of inoculated Mueller Hinton agar plates, and 40 μL of the prepared oil samples were added to the discs. Negative controls (with DMSO added onto the discs) for each tested microbe were also used. The prepared agar plates were incubated at 37 °C for 24 h, and antimicrobial efficacy was determined by gauging the diameter of the zone of inhibition (in millimetres) around the discs. Inhibition zones were measured in horizontal and vertical directions at four different places, and then the average diameter was noted. Each oil was tested in three different replicates.

#### 2.4.3. Minimum Inhibitory Concentration (MIC) Determination

Microplate turbidimetric growth inhibition assay was performed according to the method described by Pahalagedara, et al. [39] to determine the minimum concentration of oils required to inhibit bacterial growth. In brief, the oil sample (50 µL) diluted in DMSO (0.01%) was added to the 96-well microlitre plates (Thermo Fischer Scientific, Roskilde, Denmark) containing 50 µL of Mueller-Hinton broth (MHB). The overnight-grown bacterial culture was diluted, and 100 µL of this suspension was added to each well containing approximately 10^5^ cfu/mL of cells. As shown in Supplementary Figure S1, 96-well microlitre plates containing the final concentration of essential oils in each well 5, 2.5, 1.25, 0.62, 0.31, 0.16, 0.08, and 0.04% were prepared. The plates were incubated in a microplate spectrophotometer (multiskan GO, Thermo Fischer Scientific, Waltham, MA, USA) at 37 °C, and optical density was measured at a 595 nm wavelength for 24 h. An increase in optical density (turbidity) indicates bacterial growth in that well. The background correction was performed using the appropriate blanks (growth medium and oil). The plates were covered with a Breathe-Easy^®^ sealing membrane (Diversified Biotech, Dedham, MA, USA) to prevent loss of the volatiles and provide aerobic conditions to the bacteria. A control (untreated) containing only microbial suspension in the MHB was also prepared. 

### 2.5. Meat Pastes Preparation and Storage Conditions

‘Meat paste’ was chosen as a meat matrix to maintain a uniform fat distribution throughout the meat systems. Meat samples (wagyu and commercial breed beef) were cut into small cubes using a sharp knife and then passed through a mincer (Mainca, PM-98, Barcelona, Spain) with a plate of 8 mm diameter holes. The minced samples were transferred into a Hobart meat bowl chopper (Troy, NY, USA), attached with a knife and ground for about 15 min to obtain a uniform paste. Appropriate aseptic conditions were used to prepare the meat paste to avoid contamination. 

### 2.6. Preparation of Essential Oil-Added Meat Pastes

The meat paste was divided into four different lots and mixed with the bacterial culture of 10^4^–10^5^ cfu/g of *Salmonella*, *E. coli*, *L. monocytogenes*, and *S. aureus*, respectively, using a bench mixer (Kogan, 1600 W, Auckland, New Zealand). Each microbe was tested in a sterile environment on separate days to avoid cross-contamination. In addition, the container of the bench mixer (Kogan, 1600 W, New Zealand) was sterilized each time, which was used to mix meat paste and microbial cultures. The pastes were left at room temperature for 15 min to ensure the microbes’ attachment. After 10–15 min, the inoculated pastes were mixed with preservatives at a concentration of 2.5% (*v*/*w*) of MO (for Gram-negative microbes), 2.5% (*v*/*w*) of RO (for Gram-negative microbes), and 300 mg/kg of sodium nitrate (SN). However, against Gram-positive microbes, 1.25% (*v*/*w*) of MO and RO were used. A control sample for each bacteria type was also prepared and used for each meat system (commercial breed and wagyu), which contained a bacteria culture without any preservatives. Controls were used to ensure that the microbial cultures were uniformly distributed in the meat systems. All prepared samples were packed into unlaminated vacuum bags of 70-micron thickness (90 mm × 250 mm) using a vacuum packer machine and stored at a temperature of 4 °C for further analysis. Samples were removed at different time intervals of 0, 4, 10, and 16 days and analyzed for microbial growth. 

#### Microbial Growth Analysis in Meat

At selected storage time intervals, 5 g meat samples were transferred to a stomacher bag, and 45 mL of peptone water was added. The meat samples were homogenized in a stomacher bag mixer at 200 rpm for 2 min. Serial dilution of each sample was prepared and spread on selective agar plates (brilliant green modified agar for *Salmonella* spp., Eosin Methylene blue (EMB) for *E. coli*, oxford’s agar for *L. monocytogenes*, and Baird parker’s agar plates for *S. aureus*). Inoculated agar plates were incubated at 37 °C for 48 h, and colonies were enumerated. The results were expressed as log cfu/g. Each treatment was tested in triplicate meat samples. 

### 2.7. Statistical Analysis

Each analysis was performed on three different replicates (*n* = 3). To compare the effects of different treatments in the commercial breed and wagyu pastes, a statistical evaluation was carried out using a general linear model in Minitab Version 19.2020.2.0 (Minitab Inc., State College, PA, USA). The comparison was made between treatments (between mānuka oil and rosemary oil, mānuka oil and sodium nitrate, and mānuka oil and control), different meats (between wagyu and commercial breed beef pastes), and different storage periods (between different storage days). When at least one treatment was statistically different, the data were subjected to one-way analysis variance (ANOVA), followed by Tukey post hoc using the IBM^®^ SPSS^®^ Statistics software with version 22.0 (IBM Corp., Armonk, NY, USA).

## 3. Results and Discussion

### 3.1. Chemical Composition of Essential Oils

**Figure 1:**
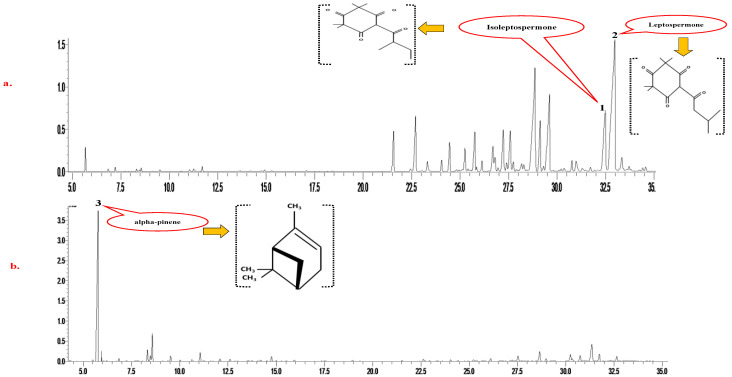
Gas chromatography-mass spectrometric (GC-MS) analysis of the (**a**) mānuka and (**b**) kānuka oils Peaks 1 and 2 in (**a**) represent the compounds isoleptospermone and leptospermone in kānuka oil 3, respectively. Peak 3 in (**b**) represents the presence of the compound alpha-pinene as a major compound in kānuka oil.

The GCMS analysis results displayed that α-pinene, β-pinene, calamene, α-terpinene, and α-terpineol are the common chemical constituents present in all essential oils. However, in MOs, the concentration of other compounds decreased as the concentration of triketones increased. The highest concentration of triketones, i.e., flavesone, leptospermone, and isoleptospermone, were found in MO 3. The GCMS results showed that the primary compounds in RO were 1, 8 cineole and α-pinene. Similarly, in KO, α-pinene was the predominant monoterpene in the highest concentrations, comprising about 60% of the composition. The significant difference in KO and MO composition was the alpha-pinene level and absence of the triketones in the latter oil in comparison with the former, as represented in Figure 1a,b. The moderate presence of sesquiterpenes, such as calamenene, viridiflorene, ledol, and viridiflorol, was also found (Supplementary Table S1). The results on the MO composition of this study agree with the study of Perry, et al. [40], who reported that the highest triketones were found in the East cape and Marlborough Sounds regions of New Zealand. Maddocks [41] also reported that all samples of KO from different locations in New Zealand contained alpha-pinene at a minimum of 60% of their volume. The chemical composition of RO has already been reported by the study of Jiang, et al. [42], in which 1, 8 cineole was the primary compound, followed by the alpha-pinene, camphor, and camphene.

### 3.2. Thermogravimetric Analysis of Essential Oils

**Figure 2:**
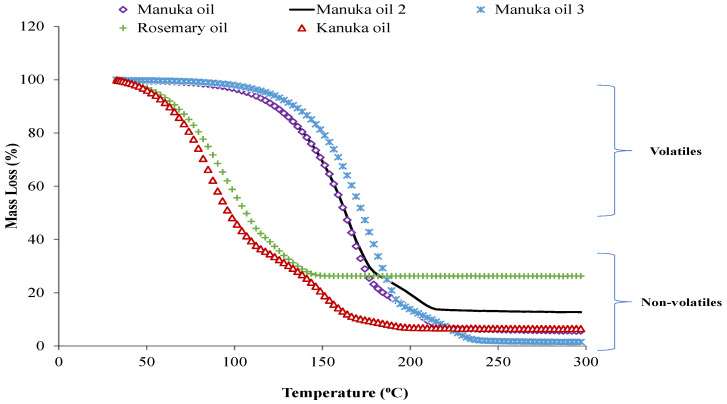
Thermogravimetric analysis of manuka 1 (5% triketones), 2 (25% triketones), 3 (40% triketones), kānuka, and rosemary oils.

The thermogravimetric weight loss of essential oil samples with an increase in temperature is shown in Figure 2. In the case of KO and RO, major weight loss was observed between 50 and 150 °C. However, the MOs were more thermostable and started losing weight after 100 °C. In the present study, the ash content of essential oils follows the order of RO > MO 2 > KO > MO 1 > MO 3. Interestingly, only MO 3 achieved the baseline (decreased to zero), while other oils showed constant weight. Herculano, et al. [43] reported that ash content could be related to the formation of heat-labile complexes between different bioactive compounds of the essential oils, which would not decompose below 300 °C. The results on the thermostability of essential oils agree with the study of Riabov, et al. [44].

The higher weight loss of RO and KOs could be attributed to the volatilization/decomposition of the bioactive compounds in essential oils, such as phenolic diterpenes [44]. Chambre, et al. [45] reported that the thermostability of essential oils could be related to their chemical composition. From the GCMS results, it can be noticed that kānuka oil contains around 50–60% of the monoterpenes, i.e., α-pinene and beta-pinene. The boiling point of alpha-pinene and beta-pinene under normal pressure is 155 °C and 165 °C, respectively. Thereby, kānuka oil lost a major part of its weight between 100–150 °C. The TGA curves of MO showed that it might be a thermostable antimicrobial agent and could have the potential to be used in cooked meat products. 

### 3.3. Antimicrobial Potential of the Essential Oils In Vitro

It can be observed from the disc diffusion assay results that the antimicrobial effects of the MOs were significantly higher than that of the KO but lower than the RO against selected Gram-negative bacteria (*Salmonella* and *E. coli*) (Table 1). RO was more effective against *Salmonella* and *E. coli* and had the highest value of inhibition zone diameter. Table 1 shows no statistically significant effect of the triketone increase against chosen Gram-negative bacteria. On the other hand, Gram-positive microbes (*L. monocytogenes* and *S. aureus*) were more sensitive to all MOs and thereby had a higher value of inhibition zone (Table 1).

Interestingly, a significant increase (*p ≤* 0.05) in the inhibition zone was observed with increased triketone content in the Mos. The MO 3 (containing the highest triketone content (40%)) produced the largest inhibition zone diameter against both tested Gram-positive bacteria. 

Figures S3–S6 show the antimicrobial effect of MO, KO, and Ros (concentration from 0.04 to 5% and time from 0 to 24 h) against *L. monocytogenes*, *S. aureus*, *Salmonella*, and *E. coli* in broths. Consistent with the disc diffusion assay results, the broth dilution assay showed that all types of MOs showed a stronger antimicrobial effect against tested Gram-positive bacteria than the RO and KO (Figures S3 and S4). For *L. monocytogenes* and *S. aureus*, at least a 0.16% concentration of MO 1 was reported as the MIC value. In MOs 2 and 3 (having 25 and 40% triketone contents, respectively), no microbial growth of either microbe was observed even at the lowest tested concentration (0.04%) (plate counting was done for this analysis, but the data is not shown). This indicates that concentrations lower than 0.04% of MO 2 and 3 can inhibit selected Gram-positive microbes’ growth. In contrast, a higher concentration (around 2.5%) of MOs was required to inhibit Gram-negative microbes, *E. coli* and *Salmonella*, than for the RO. The MIC value obtained for the RO was 2.5% (v/v) for all tested bacteria (Figures S3–S6). The antimicrobial effect of KO was less than that of MO and RO. For KO, 0.63 and 2.5% were the recorded MIC values against tested Gram-positive and Gram-negative microbes, respectively. As per the available literature, monoterpene, especially alpha-pinene, can be responsible for the antimicrobial effect of KO [24]. However, the antimicrobial potential of MO could be attributed to the presence of β-triketones, including leptospermone, flavesone, and isoleptospermone [25]. Similar to our results, van Klink, et al. [46] documented that different triketones isolated from MO were ineffective in inhibiting Gram-negative *Pseudomonas aeruginosa*. The possible explanation could be that the outer membrane of Gram-negative bacteria, made up of phospholipids, lipoproteins, lipopolysaccharides etc., may serve as a barrier, so triketones may not penetrate the cell membrane and pose any antimicrobial effect [46]. Harkenthal, Reichling, Geiss and Saller [28] have reported that the antimicrobial effect of MO was higher than that of the Australian tea tree oil, with a MIC value of 0.12% against *S. aureus*. 

Several studies have reported a varying MIC of RO against Gram-negative bacteria. Barbosa, et al. [47] reported MIC values of 0.005% (5 µL/mL) for RO against *E. coli* and 0.010 (10 µL/mL) against *Salmonella* Enteritidis. It is essential to mention that the above-mentioned studies have used different methodologies and microbial strains, which could explain the variations in the reported MIC values for the RO.

### 3.4. Essential Oil Selection for Adding in Meat Systems

Among the three MOs, only MO 2 with 25% triketone content was chosen to examine its antimicrobial effect against microbial growth in the meat systems. This selection was based on the results of preliminary experimentation involving the thermostability, antioxidant [48], and antimicrobial characteristics of these oils. As RO inhibited Gram-negative (*E. coli* and *Salmonella*) and Gram-positive bacteria (*S. aureus* and *L. monocytogenes*) at 2.5% concentration; thus, the same concentration of MO was selected to compare their antibacterial effect. However, due to the strong antibacterial effect of MO against *L. monocytogenes* and *S. aureus* (0.04% (confirmed through broth dilution assay)), lower concentrations of MO can be employed to prevent these microbes. KO had lower antioxidant and antimicrobial properties than mānuka oil, so it was not examined in meat pastes.

Regarding the chemical composition of the meat pastes, wagyu paste contains 64.8% moisture, 12.3% fat, and 19.4% protein. However, commercial beef paste had lower fat content (3.4%) and higher- moisture content than wagyu beef, as reported by our recent paper [48]. The moisture content of the meat paste samples was checked according to the method of AOAC [49]. The Dumas method [49] was used to determine the protein content. The obtained values were multiplied with a factor of 6.25 to calculate protein content. For the estimation of fat content, the Soxhlet method was used [49,50,51].

#### Effects of the Essential Oils against Microbial Growth in Meat Pastes

The changes in microbial growth in wagyu and commercial breed meat paste with or without any added preservative agent during storage at 4 °C for 16 days are presented in Table 2. 

In both types of meat pastes, MO treatment significantly inhibited the growth of Gram-positive microbes during storage, followed by RO and SN. The treatment of commercial breed pastes with MO resulted in *L. monocytogenes* and *S. aureus* counts lowering by 3 and 2.9 log cfu/g, respectively, compared to the control. However, in wagyu paste, the inhibition effect of MO showed reduced growth of *S. aureus* (1.6 log cfu/g) and *L. monocytogenes* (2.5 log cfu/g) when compared to their control counterparts. The addition of SN to wagyu meat matrix samples resulted in 0.12 log cfu/g populations of *E. coli*, lower than their control samples.

On the other hand, RO was most effective against Gram-negative microbes and showed higher antimicrobial efficacy than the MO and SN-treated meat pastes. In commercial breed meat pastes, treatment with RO resulted in 1.67 log cfu/g of *E. coli*, whereas 0.77 log cfu/g of *E. coli* was found with MO and 0.12 log cfu/g of *E. coli* was found with SN treatments; both lower than the control. However, this inhibition was less pronounced in wagyu meat, showing 1.19 log cfu/g of *E. coli*, lower than their control counterparts (Table 2).

The lower antimicrobial effect of the MO against Gram-negative bacteria has already been reported through in vitro studies [46]. However, previous research studies on the antimicrobial potential of RO against *Salmonella* and *E. coli* in meat models exhibited variable results. Ahn, et al. [52] showed approximately a one log reduction in *E. coli*, *Salmonella*, and *L. monocytogenes* counts with 1% rosemary oleoresin in ground beef stored under refrigerated conditions (4 °C) for nine days. Stojanović-Radić, et al. [53] reported that a shorter exposure (soaking) time (~15 min) with rosemary oil exhibited a higher antimicrobial effect against *Salmonella* Enteritidis, in thermally processed chicken meat, than the untreated control. However, Kahraman, et al. [54] did not observe any antimicrobial effect of the rosemary oil (0.2% concentration) against *L. monocytogenes* and *Salmonella* Typhimurium in poultry fillets. Although in our study, the effect was similar to or higher than the previous studies, it is possibly due to various factors such as the composition and concentration of the oils, different meat types (pork, beef or chicken), and inoculum size and sensitivity, which influenced the experimental results. 

Comparing the antimicrobial effect of RO and MO, a significant difference in their antimicrobial activity was found, which could be attributed to their chemical constituents 1, 8-cineole and β-triketones, respectively. Due to these compounds’ hydrophobic nature, they can rupture bacterial cell membranes and damage microbial cells’ vital functions [16]. However, the presence of a hydroxyl group in the structure of phenolic compounds, such as carvacrol, a bioactive compound in RO, makes them more strongly active than esters and hydrocarbons. It has been reported that -OH groups can easily form hydrogen bonds with the active site of enzymes [55].

Interestingly, the compositional difference of both meat pastes significantly affected the microbial growth in all treated samples, especially in control samples. The probable reasons could be a difference in fat content and fatty acid composition of both meat products [56]. Secondly, as both meat pastes contain different fat and moisture content, there could be differences in the water activity and, thereby, the growth of microbes [57]. 

As per our results, the wagyu paste (high-fat meat matrix) treated with oils (either MO or RO) exhibited a lower inhibition of microbial growth than the commercial breed paste (low-fat meat matrix). It can be related to the influence of fat or the octanol-water partition coefficient of the major constituents of the oils. The octanol-water partition coefficients of the main compounds in MO and RO were above 4 (data not shown). Exhibiting that these compounds will be more present in the fat part of the meat than in the aqueous phase. Their retention in non-aqueous food phases might not show the desired antimicrobial effect and direct action on the targeted microbe in the aqueous phase [58]. Owing to the absorption of carvacrol in the fat of regular ground beef, a decreased antimicrobial activity of carvacrol in regular beef (12% fat) than in lean beef (5% fat) has been reported by Wang, Heising, Fogliano and Dekker [32]. Similarly, in low and zero-fat hotdogs, thyme oil reduced the *L. monocytogenes* numbers but did not show any appropriate effect in full-fat hotdogs [31]. Our study’s findings support that the fat content of meat and meat products may influence the antimicrobial efficacy of essential oils. 

This study showed the antimicrobial potential of mānuka and rosemary oils against selected pathogenic microbes; as antimicrobial susceptibility may vary between strains in the same bacterial species, thus these oils need to be tested against several strains from each species to provide concrete evidence of their antimicrobial activity against the targeted microbial species. It is important to mention that the sensory analysis of mānuka and rosemary oils to added beef pastes is essential for consumer acceptability; this analysis may be considered in future work. Additionally, studies of the safety and toxicity analysis of mānuka and kānuka oils will be needed to ensure their safety and acceptability for food applications.

## 4. Conclusions

In conclusion, this study revealed that all MOs (triketone contents 5, 25, and 40%) exerted better antimicrobial activities than KO against Gram-negative and Gram-positive microbes, as exhibited by the in vitro results. As per the disc diffusion and broth dilution assay results, *L. monocytogenes* and *S. aureus* exhibited more sensitivity to MOs than Gram-negative microbes. Increased antimicrobial efficacies were observed with increased triketone content of MOs against selected Gram-positive bacteria (*L. monocytogenes* and *S. aureus*). However, RO inhibited *Salmonella* and *E. coli* more effectively than the KO and MOs.

In commercial breed (low-fat meat system) and wagyu (high-fat meat system) meat pastes, MO showed a significantly greater inhibitory effect against Gram-positive bacteria than the RO- and SN-treatments. However, RO lowered the growth of Gram-negative microbes more effectively than all other treated samples. Therefore, the results indicate that MO is prefered for use in food products as a natural antimicrobial agent against *L. monocytogenes* and *S. aureus* at low concentrations when compared to RO, while RO can be used against *Salmonella* and *E. coli* (at high concentrations). In the future, research on encapsulation and emulsification of these oils can ameliorate their stability and solubility, mask undesirable effects (taste and odour) and facilitate a wide range of food applications. In addition, future studies on these oils’ toxicity effects are required to ensure their safety and acceptability for human consumption before food applications. From a food application viewpoint, sensory approaches to essential oils-added food products will provide concrete proof of their acceptability in foods.

## Figures and Tables

**Table 1:** The inhibition zone values were observed for mānuka, kanuka, and rosemary oils against different microorganisms.

Oil Type	Zone of Inhibition (mm)			
	*Listeria monocytogenes*	*Staphylococcus aureus*	*Escherichia coli*	*Salmonella* spp.
Rosemary oil	6.9 ± 0.5 ^e^	6.2 ± 0.3 ^e^	17.5 ± 0.5 ^a^	18.27 ± 1.6 ^a^
Kānuka oil	9.83 ± 0.2 ^d^	9.7 ± 0.5 ^d^	11.43 ± 0.5 ^c^	14.17 ± 0.7 ^b^
Mānuka oil 1 (5% Triketone content)	17.53 ± 0.5 ^c^	11.38 ± 0.3 ^c^	11.27 ± 1.1 ^c^	14.2 ± 0.7 ^b^
Mānuka oil 2 (25% Triketone content)	23.5 ± 0.5 ^b^	16.01 ± 0.1 ^b^	14.06 ± 1.0 ^b^	15.1 ± 0.8 ^b^
Mānuka oil 3 (40% Triketone content)	26 ± 0.2 ^a^	17.69 ± 0.4 ^a^	14.27 ± 1.1 ^b^	15.3 ± 0.5 ^b^

Different superscripts within a column represent a statistically significant difference (*p ≤* 0.05).

**Table 2:** The changes in microbial growth in wagyu and commercial breed meat matrices with or without any added preservative agent during storage at 4 °C for 16 days.

	Meat System	Treatments				SEM	*p*-Value		
		MO	RO	SN	C		MO × RO	MO × SN	MO × C
***Staphylococcus aureus***									
0	Wagyu	5.69 ^Xa^	5.68 ^Ya^	5.68 ^Xa^	5.71 ^Xa^	0.01	*p* > 0.05	*p* > 0.05	*p* > 0.05
	Commercial	5.67 ^Xb^	5.83 ^Xa^	5.77 ^Xab^	5.88 ^Xa^	0.02	*p* < 0.05	*p* > 0.05	*p* < 0.05
4	Wagyu	6.01 ^Xb^	6.18 ^Xb^	6.45 ^Xb^	6.91 ^Xa^	0.09	*p* < 0.05	*p* < 0.01	*p* < 0.05
	Commercial	5.70 ^Yb^	5.69 ^Yb^	5.82 ^Yb^	6.40 ^Xa^	0.08	*p* < 0.01	*p* > 0.05	*p* < 0.05
10	Wagyu	6.53 ^Xd^	6.63 ^Xc^	6.84 ^Xb^	7.47 ^Xa^	0.01	*p* < 0.01	*p* < 0.01	*p* < 0.01
	Commercial	5.11 ^Yd^	5.37 ^Yc^	6.32 ^Yb^	7.27 ^Xa^	0.01	*p* < 0.01	*p* < 0.01	*p* < 0.01
16	Wagyu	5.93 ^Xd^	6.23 ^Xc^	6.86 ^Xb^	7.46 ^Ya^	0.02	*p* < 0.01	*p* < 0.01	*p* < 0.01
	Commercial	5.08 ^Yd^	5.54 ^Yc^	6.32 ^Yb^	7.90 ^Xa^	0.04	*p* < 0.01	*p* < 0.01	*p* < 0.01
SE	Wagyu	*p* < 0.05	*p* < 0.05	*p* < 0.05	*p* < 0.05				
	Commercial	*p* < 0.05	*p* < 0.05	*p* < 0.05	*p* < 0.05				
***Listeria monocytogenes***									
0	Wagyu	5.83 ^Xb^	5.85 ^Xb^	5.84 ^Xb^	5.96 ^Xa^	0.10	*p* > 0.05	*p* > 0.05	*p* < 0.01
	Commercial	5.66 ^Yd^	5.75 ^Yc^	5.83 ^Xb^	5.92 ^Ya^	0.01	*p* < 0.05	*p* < 0.01	*p* < 0.01
4	Wagyu	6.69 ^Xd^	6.89 ^Xc^	7.45 ^Xa^	7.12 ^Yb^	0.02	*p* < 0.05	*p* < 0.01	*p* < 0.01
	Commercial	6.67 ^Yd^	6.87 ^Xc^	7.34 ^Yb^	7.53 ^Xa^	0.80	*p* < 0.01	*p* < 0.01	*p* < 0.01
10	Wagyu	6.47 ^Xd^	6.70 ^Xc^	7.57 ^Yb^	7.89 ^Ya^	0.02	*p* < 0.01	*p* < 0.01	*p* < 0.01
	Commercial	6.41 ^Yd^	6.67 ^Xc^	7.63 ^Xb^	8.18 ^Xa^	0.01	*p* < 0.01	*p* < 0.01	*p* < 0.01
16	Wagyu	6.04 ^Xd^	6.45 ^Xc^	7.71 ^Yb^	8.57 ^Ya^	0.02	*p* < 0.01	*p* < 0.01	*p* < 0.01
	Commercial	5.98 ^Xd^	6.45 ^Xc^	7.88 ^Xb^	8.94 ^Xa^	0.03	*p* < 0.01	*p* < 0.01	*p* < 0.01
SE	Wagyu	*p* < 0.05	*p* < 0.05	*p* < 0.05	*p* < 0.05				
	Commercial	*p* < 0.05	*p* < 0.05	*p* < 0.05	*p* < 0.05				
***Escherichia coli***									
0	Wagyu	5.82 ^Xab^	5.66 ^Xc^	5.75 ^Xb^	5.85 ^Xa^	0.01	*p* > 0.05	*p* < 0.05	*p* < 0.05
	Commercial	5.58 ^Yb^	5.62 ^Xab^	5.70 ^Xa^	5.70 ^Ya^	0.01	*p* > 0.05	*p* < 0.05	*p* < 0.01
4	Wagyu	6.41 ^Yc^	6.42 ^Yc^	6.51 ^Yb^	7.54 ^Xa^	0.01	*p* > 0.05	*p* < 0.01	*p* < 0.01
	Commercial	7.02 ^Xc^	5.25 ^Xd^	7.40 ^Xb^	7.49 ^Ya^		*p* < 0.01	*p* < 0.01	*p* < 0.01
10	Wagyu	7.41 ^Xc^	6.41 ^Yd^	8.15 ^Xb^	8.68 ^Xa^	0.00	*p* < 0.01	*p* < 0.01	*p* < 0.01
	Commercial	7.41 ^Xb^	6.61 ^Xd^	7.33 ^Yc^	7.71 ^Ya^	0.00	*p* < 0.01	*p* < 0.01	*p* < 0.01
16	Wagyu	8.15 ^Xc^	6.54 ^Yd^	8.64 ^Yb^	9.34 ^Xa^	0.08	*p* < 0.01	*p* > 0.05	*p* < 0.01
	Commercial	8.53 ^Xb^	7.63 ^Xc^	9.18 ^Xab^	9.30 ^Xa^	0.14	*p* < 0.01	*p* < 0.01	*p* > 0.05
SE	Wagyu	*p* < 0.01	*p* < 0.01	*p* < 0.01	*p* < 0.01				
	Commercial	*p* < 0.01	*p* < 0.01	*p* < 0.01	*p* < 0.01				
***Salmonella* spp.**									
0	Wagyu	5.75 ^Xb^	5.62 ^Yc^	5.70 ^Xb^	5.86 ^Xa^	0.01	*p* < 0.01	*p* > 0.05	*p* < 0.01
	Commercial	5.73 ^Xb^	5.70 ^Xb^	5.75 ^Xb^	5.81 ^Xa^	0.03	*p* > 0.05	*p* > 0.05	*p* < 0.01
4	Wagyu	6.81 ^Yab^	5.71 ^Xc^	6.62 ^Yb^	7.25 ^Xa^	0.1	*p* < 0.01	*p* < 0.01	*p* > 0.05
	Commercial	6.90 ^Xa^	5.84 ^Xb^	6.72 ^Xa^	7.20 ^Xa^	0.10	*p* < 0.01	*p* < 0.01	*p* > 0.05
10	Wagyu	7.65 ^Yb^	6.42 ^Xd^	7.45 ^Xc^	8.40 ^Xa^	0.09	*p* < 0.01	*p* < 0.01	*p* < 0.01
	Commercial	8.20 ^Xb^	5.95 ^Yd^	7.59 ^Yc^	8.58 ^Xa^	0.11	*p* < 0.01	*p* < 0.01	*p* < 0.01
16	Wagyu	8.12 ^Xb^	6.24 ^Xd^	7.68 ^Xc^	8.69 ^Xa^	0.01	*p* < 0.01	*p* < 0.01	*p* < 0.01
	Commercial	7.40 ^Yb^	5.16 ^Yc^	7.50 ^Yb^	8.46 ^Xa^	0.15	*p* < 0.05	*p* < 0.01	*p* < 0.01
SE	Wagyu	*p* < 0.01	*p* < 0.01	*p* < 0.01	*p* < 0.01				
	Commercial	*p* < 0.01	*p* < 0.01	*p* < 0.01	*p* < 0.01				

Treatments—MO—Mānuka oil, RO—Rosemary oil, SN—Sodium Nitrate, and C—Control. MO × RO = comparison between mānuka oil and rosemary oil, MO × SN = comparison between mānuka oil and sodium nitrate, MO × C = comparison between mānuka oil and control, SE = Storage effect, and Storage effect (0th × 7th day) = comparison between 0th and 7th day. SEM—Standard error mean. ^a–d^ Means within a row with the same superscript letters are not significantly different (*p* < 0.05) between the treatments on the same storage day. ^XY^ Means within a column with the same superscript letters are not significantly different (*p* < 0.05) between the meat systems

## Data Availability

Data will be made available upon request.

## Supplementary Materials

The following supporting information can be downloaded at: https://www.mdpi.com/article/10.3390/foods12061333/s1. Figure S1. The layout of the 96-well plate experiment used for the broth dilution method. Figure S2. Zone of inhibition formed by mānuka (1, 2 and 3 with 5, 25 and 40% triketones, respectively), kānuka and rosemary oils against *Listeria monocytogenes.* Figure S3 *Staphylococcus aureus* growth curves for (A) rosemary oil; (B) kānuka oil; (C) mānuka oil 1 (5% triketone content); (D) mānuka oil 2 (25% triketone content); and (E) mānuka oil 3 (40% triketone content) at different concentrations. (The control sample contained no preservatives, but the test organism *Staphylococcus aureus* in Mueller-Hinton broth). The antimicrobial effects of all essential oils were determined at concentrations ranging from 5 to 0.04 %. However, only selected concentrations showing major differences are presented in Figures S3–S6. Figure S4 *Listeria monocytogenes* growth curves for (a) rosemary oil; (b) kānuka oil; (c) mānuka oil 1 (5% triketone content); (d) mānuka oil 2 (25 % triketone content); and (e) mānuka oil 3 (40% triketone content) at different concentrations (The control sample contained no preservative, but the test organism *Listeria monocytogenes* in Mueller-Hinton broth). Figure S5 *Salmonella* spp. growth curves for (A) rosemary oil; (B) kānuka oil; (C) mānuka oil 1 (5% triketone content); (D) mānuka oil 2 (25% triketone content); and (E) mānuka oil 3 (40% triketone content) at different concentrations (The control sample contained no preservative, but the test organism *Salmonella* spp. in Mueller-Hinton broth). Figure S6 *Escherichia coli* growth curves for (a) rosemary oil; (b) kānuka oil; (c) mānuka oil 1 (5% triketone content); (d) mānuka oil 2 (25% triketone content); and (e) mānuka oil 3 (40% triketone content) at different concentrations (The control sample contained no preservatives, but the test organism *Escherichia coli* in Mueller-Hinton broth). Table S1. Main constituents in mānuka, kānuka and rosemary oil identified through gas chromatography and mass spectrometry analysis.
