# Supplementary material for: Mānuka Oil vs. Rosemary Oil: Antimicrobial Efficacies in Wagyu and Commercial Beef against Selected Pathogenic Microbes
Source: Foods. 2023 Mar 21;12(6):1333. doi: 10.3390/foods12061333 (PMC10048739; doi:10.3390/foods12061333)
Supplement: Supplementary file 1 [file foods-12-01333-s001.zip › foods-2247613-supplementary.pdf]

foods
MDPI

Supplementary data

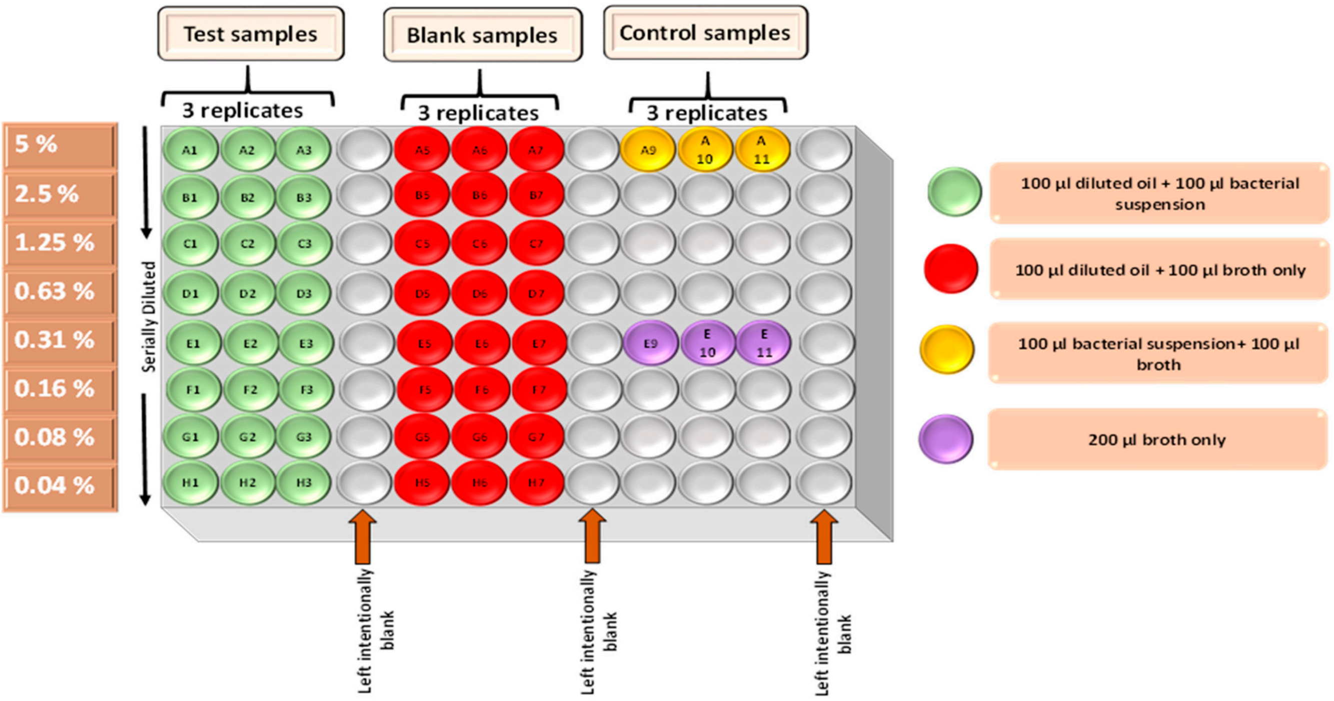

Figure S1. The layout of the 96-well plate experiment used for the broth dilution method.

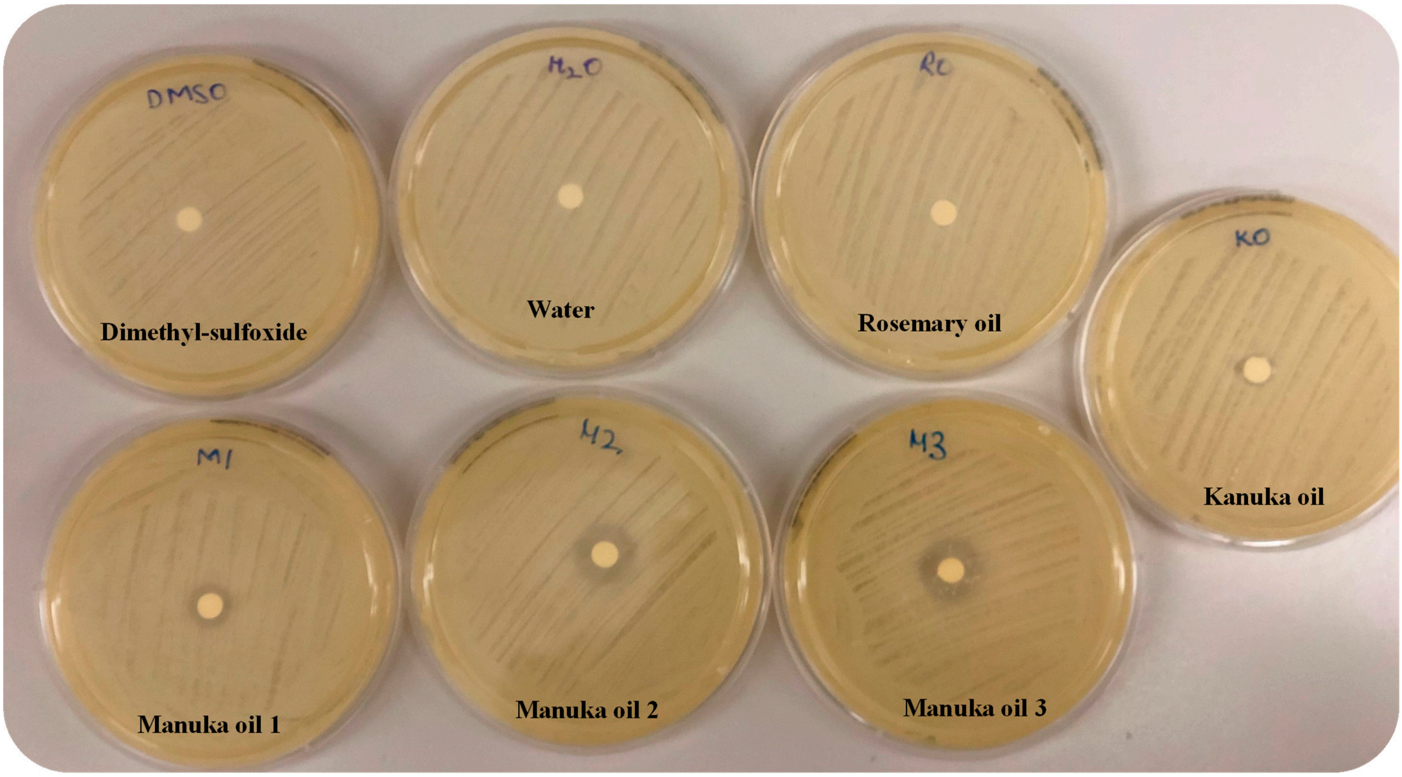

Figure S2. Zone of inhibition formed by mānuka (1,2 and 3 with 5, 25 and 40 % triketones, respectively), kānuka and rosemary oils against ***Listeria monocytogenes*****.**

Activity against targeted microbial species.

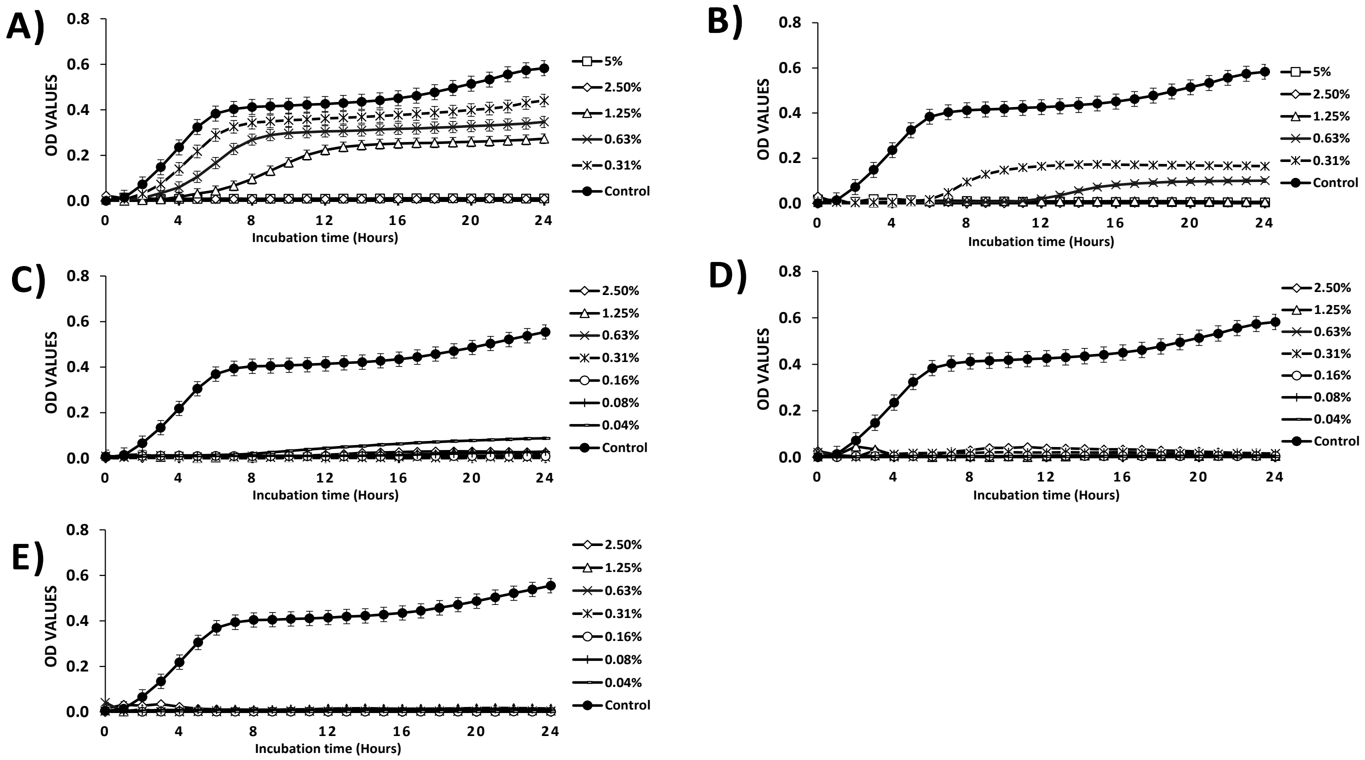

Figure S3 *Staphylococcus aureus* growth curves for A) rosemary oil; B) kānuka oil; C) mānuka oil 1 (5 % triketone content); D) mānuka oil 2 (25 % triketone content); and E) mānuka oil 3 (40 % triketone content) at different concentrations. (The control sample contained no preservatives, but the test organism *Staphylococcus aureus* in Mueller-Hinton broth). The control sample contained no preservative but the test organism, *Staphylococcus aureus*. The antimicrobial effects of all essential oils were determined at concentrations ranging from 5 to 0.04 %. However, only selected concentrations showing major differences are presented in Figures 3, 4, 5 and 6.

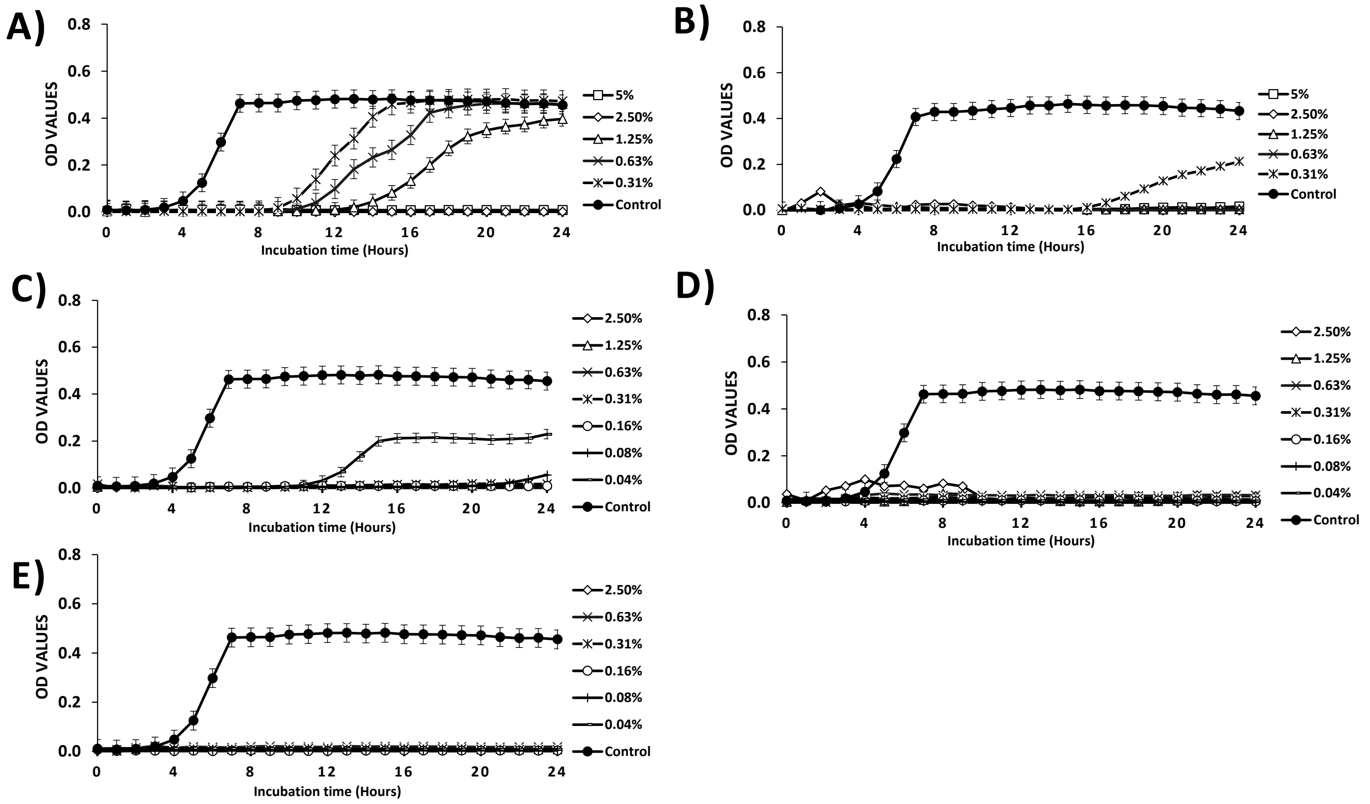

Figure S4 *Listeria monocytogenes* growth curves for a) rosemary oil; b) kānuka oil; c) mānuka oil 1 (5 % triketone content); d) mānuka oil 2 (25 % triketone content); and e) mānuka oil 3 (40 % triketone content) at different concentrations. (The control sample contained no preservative, but the test organism *Listeria monocytogenes* in Mueller-Hinton broth).

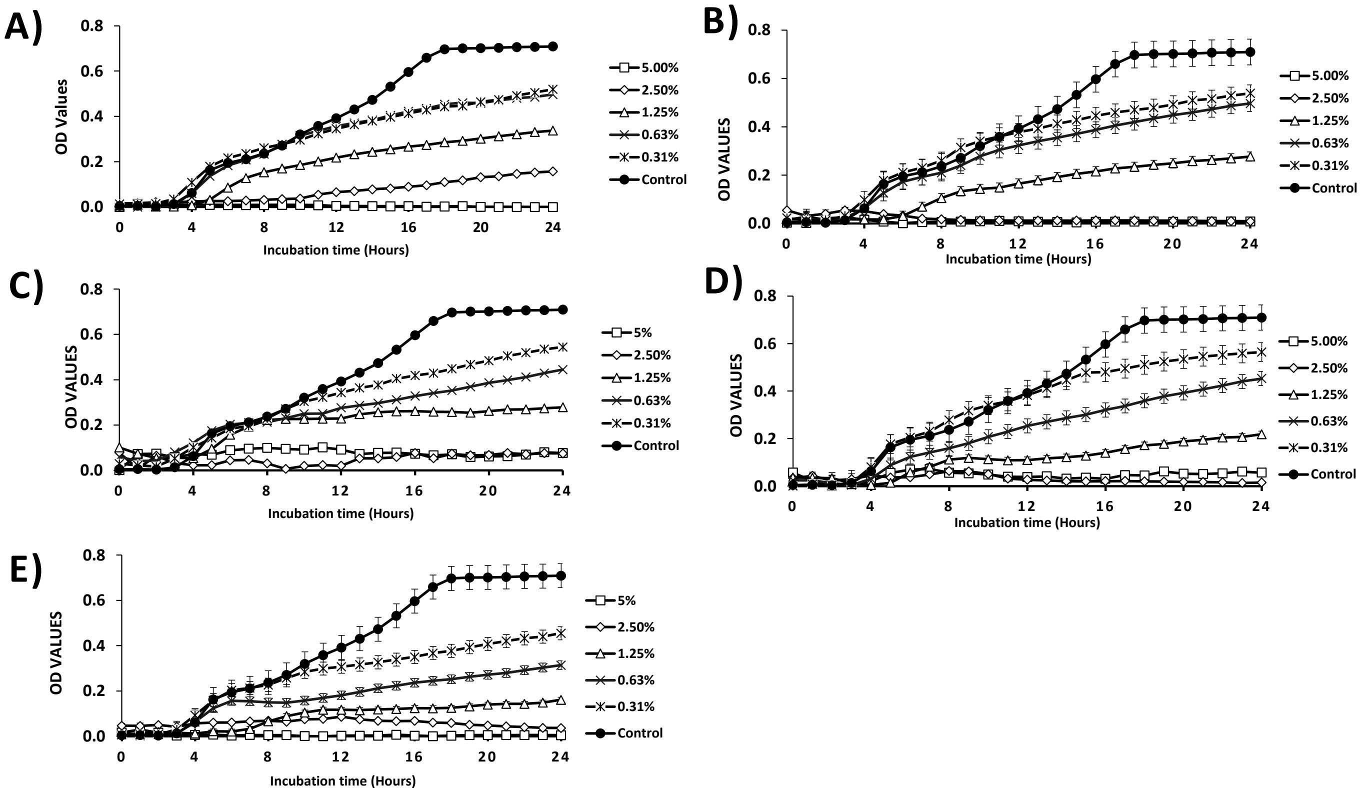

Figure S5 *Salmonella* spp. growth curves for A) rosemary oil; B) kānuka oil; C) mānuka oil 1 (5 % triketone content); D) mānuka oil 2 (25 % triketone content); and E) mānuka oil 3 (40 % triketone content) at different concentrations. (The control sample contained no preservative, but the test organism *Salmonella* spp in Mueller-Hinton broth).

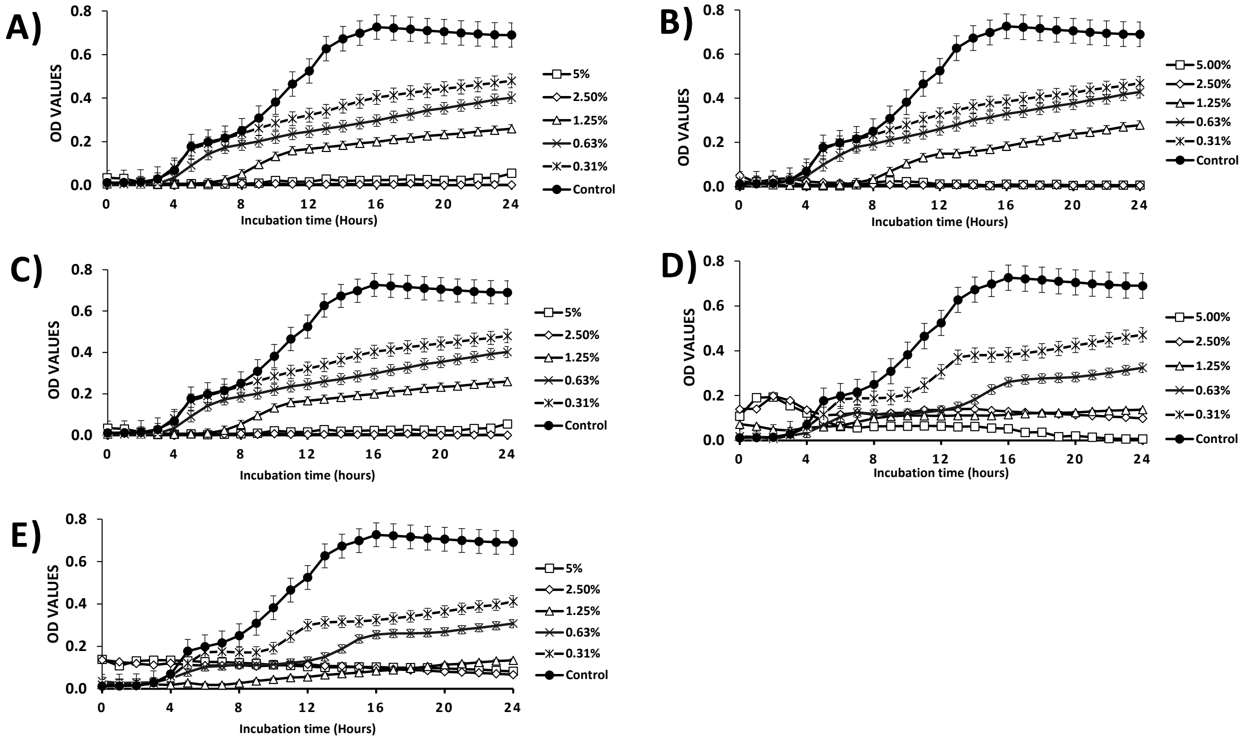

Figure S6 *Escherichia coli* growth curves for a) rosemary oil; b) kānuka oil; c) mānuka oil 1 (5 % triketone content); d) mānuka oil 2 (25 % triketone content); and e) mānuka oil 3 (40 % triketone content) at different concentrations. (The control sample contained no preservatives, but the test organism *Escherichia coli* in Mueller-Hinton broth).

Table S1. Main constituents in mānuka, kānuka and rosemary oil identified through gas chromatography and mass spectrometry analysis.

Sr No	Chemical compound name	Mānuka oil 1 (Area %)	Mānuka oil 2 (Area %)	Mānuka oil 3 (Area %)	Rosemary oil (Area %)	Kānukaoil (Area %)
1.	**α-pinene**	**1.32**	**1.19**	**0.92**	**19.09**	**64.25**
2.	**β-Pinene**	**0.37**	**0.17**	**0.15**	**5.31**	**0.71**
3.	**β -Myrcene**	**0.35**	**0.3**	**0.27**	**3.14**	**-**
4.	**Γ-terpinene**	**0.44**	**0.15**	**0.14**	**2.34**	**1.52**
5.	**Limonene**	**0.7**	**0.12**	**0.1**	**0.2**	**1.79**
6.	**linalool**	**-**	**-**	**-**	**2.91**	**2.76**
7.	**α-terpinol**	**0.21**	**0.2**	**0.22**	**-**	**-**

**8.**	**p- Cymene**	**0.44**	**0.08**	**0.15**	**-**	**3.28**
**9.**	**1,8 cineole**	**0.35**	**0.23**	**0.22**	**50.75**	**6.6**
**10.**	**β -Cryophyllene**	**3.7**	**2.56**	**2.4**	**6.46**	**-**
**11.**	**Aromadendrene**	**2.51**	**2.24**	**1.85**	**-**	**-**
**12.**	**α-gurjuene**	**0.32**	**0.26**	**0.24**	**-**	**-**
**13.**	**δ-cardinene**	**8.26**	**5.49**	**6.1**	**-**	**-**
**14.**	**Alloaromadendrene**	**1.16**	**0.96**	**0.81**	**-**	**-**
**15.**	**Cardia- 3,9 diene**	**4.15**	**4.97**	**4.67**	**-**	**-**
**16.**	**B-elemene**	**0.4**	**0.39**	**0.38**	**-**	**-**
**17.**	**α-farnesene**	**1.75**	**2.2**	**2.04**	**-**	**-**
**18.**	**α-cedrene**	**6.05**	**4.39**	**4.22**	**-**	**-**
**19.**	**Calamene or cardia 1,4 dinene**	**16.42**	**13.23**	**11.01**	**-**	**3.78**
**20.**	**Flavesone**	**1.89**	**6.26**	**8.57**	**-**	**-**
**21.**	**γ-Elemene**	**1.73**	**1.33**	**1.28**	**-**	**-**
**22.**	**β-Selinene**	**5.26**	**0.08**	**0.92**	**-**	**-**
**23.**	**α-Cubebene**	**4.88**	**3.59**	**3.41**	**-**	**-**
**24.**	**Ylangene**	**0.36**	**0.3**	**0.26**	**-**	**-**
**25.**	**Copaene**	**6.01**	**4.88**	**4.19**	**-**	**-**
**26.**	**cubenol**	**0.65**	**0.17**	**0.13**	**-**	**-**
**27.**	**globulul**	**0.49**	**0.28**	**0.22**	**-**	**-**
**28.**	**leptospermone**	**1.09**	**5.19**	**4.89**	**-**	**-**
**29.**	**isoleptospermone**	**3.89**	**12.37**	**18.39**	**-**	**-**
**30.**	**verdiflorol**	**2.61**	**2.41**	**1.94**	**-**	**8**
**31.**	**spanthul**	**1.23**	**1.24**	**1.11**	**-**	**2.53**
**32.**	**Grandiflorone**	**-**	**0.23**	**0.56**	**-**	
**33.**	**aliphatic ester**	**-**	**0.23**	**0.18**	**-**	
**34.**	**n-Amyl isovalerate**		**0.1**	**0.3**		**-**
**35.**	**α-thujene**	**-**	**-**	**-**	**-**	**0.68**

**36.**	**Nerolidol**	**-**	**-**	**-**	**-**	**2.53**
